# How Can Childbirth Care for the Rural Poor Be Improved? A Contribution from Spatial Modelling in Rural Tanzania

**DOI:** 10.1371/journal.pone.0139460

**Published:** 2015-09-30

**Authors:** Piera Fogliati, Manuela Straneo, Cosimo Brogi, Pier Lorenzo Fantozzi, Robert Mahimbo Salim, Hamis Mwendo Msengi, Gaetano Azzimonti, Giovanni Putoto

**Affiliations:** 1 Doctors with Africa–CUAMM, Padua, Italy; 2 Doctors with Africa–CUAMM, Iringa, Tanzania; 3 Department of Physical Sciences, Earth and Environment, University of Siena, Siena, Italy; 4 Regional Medical Office, Iringa Region, Iringa, Tanzania; 5 Council Medical Office, Ludewa District Council, Ludewa, Tanzania; Centers for Disease Control and Prevention, UNITED STATES

## Abstract

**Introduction:**

Maternal and perinatal mortality remain a challenge in resource-limited countries, particularly among the rural poor. To save lives at birth health facility delivery is recommended. However, increasing coverage of institutional deliveries may not translate into mortality reduction if shortage of qualified staff and lack of enabling working conditions affect quality of services. In Tanzania childbirth care is available in all facilities; yet maternal and newborn mortality are high. The study aimed to assess in a high facility density rural context whether a health system organization with fewer delivery sites is feasible in terms of population access.

**Methods:**

Data on health facilities’ location, staffing and delivery caseload were examined in Ludewa and Iringa Districts, Southern Tanzania. Geospatial raster and network analysis were performed to estimate access to obstetric services in walking time. The present geographical accessibility was compared to a theoretical scenario with a 40% reduction of delivery sites.

**Results:**

About half of first-line health facilities had insufficient staff to offer full-time obstetric services (45.7% in Iringa and 78.8% in Ludewa District). Yearly delivery caseload at first-line health facilities was low, with less than 100 deliveries in 48/70 and 43/52 facilities in Iringa and Ludewa District respectively. Wide geographical overlaps of facility catchment areas were observed. In Iringa 54% of the population was within 1-hour walking distance from the nearest facility and 87.8% within 2 hours, in Ludewa, the percentages were 39.9% and 82.3%. With a 40% reduction of delivery sites, approximately 80% of population will still be within 2 hours’ walking time.

**Conclusions:**

Our findings from spatial modelling in a high facility density context indicate that reducing delivery sites by 40% will decrease population access within 2 hours by 7%. Focused efforts on fewer delivery sites might assist strengthening delivery services in resource-limited settings.

## Introduction

Maternal deaths worldwide remain a major public health issue, with 289 000 deaths from complications of pregnancy and delivery in 2013 [1]. More than half occur in sub-Saharan Africa, with the highest mortality ratios in rural areas and among poorer communities [2]. The picture of this tragedy is completed by newborn outcomes; about three million neonates die every year and an additional 2.6 million are stillborn [3]. Not unexpectedly maternal health and newborn health are closely linked and deaths are preventable if adequate and timely childbirth care is provided by skilled health personnel based in functioning health facilities [4].

Improving institutional delivery coverage is one strategy advocated to reduce deaths among the rural poor. Although proximity to health facility is strongly associated with higher facility births [5], the mere facility use for delivery does not translate into early neonatal or maternal mortality reduction [6]. Shorter distance to emergency obstetric and neonatal care is associated with lower early neonatal mortality only if high level of care is provided [7, 8]. In other words, mortality during childbirth depends on factors related to the quality of services offered, such as the 24 hours/7 days availability of qualified personnel supported by expertise, medical supplies, drugs and by a functioning referral system.

With a mortality rate of 454 per 100,000 live births in 2010, the United Republic of Tanzania is unlikely to meet the Millennium Development Goal target of 218 deaths for 100,000 live births by 2015 [9]. Tanzania is a low-resources country with a pyramidal-shaped health care system. First-line facilities, namely dispensaries and health centres are at the base offering primary level care and referral hospitals at the apex. Basic childbirth services are provided at all levels while obstetric interventions including surgery and blood transfusion are generally only available in district or higher level hospitals [10]. To improve population coverage an increased number of first-line facilities is planned [11] and to reduce maternal, newborn mortality and morbidity the majority of first-line facilities are set to provide basic emergency obstetric and neonatal care [12, 13]. Obstetric services are classified according to the level of care provided to treat obstetric complications in Basic Emergency Obstetric Care (BEmOC) and Comprehensive Emergency Obstetric Care (CEmOC). To qualify as BEmOC health facilities have to regularly perform seven signal functions (administration of parental antibiotics, uterotonic drugs, and anticonvulsants, manual removal of placenta, removal of retained products, assisted vaginal deliveries, and neonatal resuscitation) whereas CEmOC carry out also caesarean sections and blood transfusions [14].

Poorly equipped, understaffed first-line health facilities with low delivery caseload have been described in rural Tanzania and are considered a major barrier to quality childbirth care [15–18]. Rural poor are disadvantaged in accessing quality services compared to wealthier women as they are less likely to bypass first-line facilities to deliver at high volume and high quality hospital level [18, 19]. Recent studies have suggested that in a scenario of increasing health facility density and persisting limited resources, childbirth care for the rural population could be improved by concentrating available resources in fewer delivery sites without modifying facilities numbers [18, 19]. Delivery sites should have good geographical accessibility and be adequately equipped and staffed to provide maternal services 24h/7d, while remaining first-line health facilities would continue to provide other preventive and curative services. The question that arises is whether delivery site reduction may compromise population accessibility.

In an attempt to define a health system reorganization with delivery sites, factors as geographical accessibility, population density, transport and means of communication must be taken into consideration. Although distance has been traditionally used as a measure of physical accessibility, travel time to reach BEmOC facilities has become a more accurate indicator for monitoring maternal mortality reduction interventions, especially in rural areas where lack of transport and geographical barriers might delay access to life-saving services. A maximum of two hours’ travel time has been indicated to reach BEmOC services [20]. This is the time available to treat haemorrhage, the most rapidly fatal complication of pregnancy, and basic obstetric services should be accessible to the majority of women within this time span [21].

Geographical Information System (GIS) technology and spatial modelling can play a key role in public health, particularly in assessing physical access to health services and planning resource allocation [22, 23]. The application of raster and network methods for estimating distance and travel time in health services research has been extensively described [24]. Raster methods are mostly used for rural areas with limited infrastructure while network methods are suitable for urban settings with road-connected health facilities. Spatial analysis based on network methods is considered more accurate than raster methods as it relies on existing paths rather than Euclidean distances [25].

The study aimed to assess whether a health system organization with fewer delivery sites is possible in terms of population access within 2 hours’ walking time. We carried out the research in a rural context in Southern Tanzania characterized by a high coverage of institutional deliveries, high health facility density and limited infrastructure network [26]. We investigated whether and to what extend a reduction of delivery sites was feasible to allow access within 2 hours’ travel time to about 80% of the population, in line with the Tanzania target of 80% skilled birth attendance (SBA) coverage by end 2015 [27]. To estimate walking time in rural areas we tested an innovative network-based approach and we compared it with a raster method for validation.

The findings from this study will provide a new perspective for policymakers on how to organize childbirth services in resource-limited settings.

## Methods

### Study area

**Fig 1:**
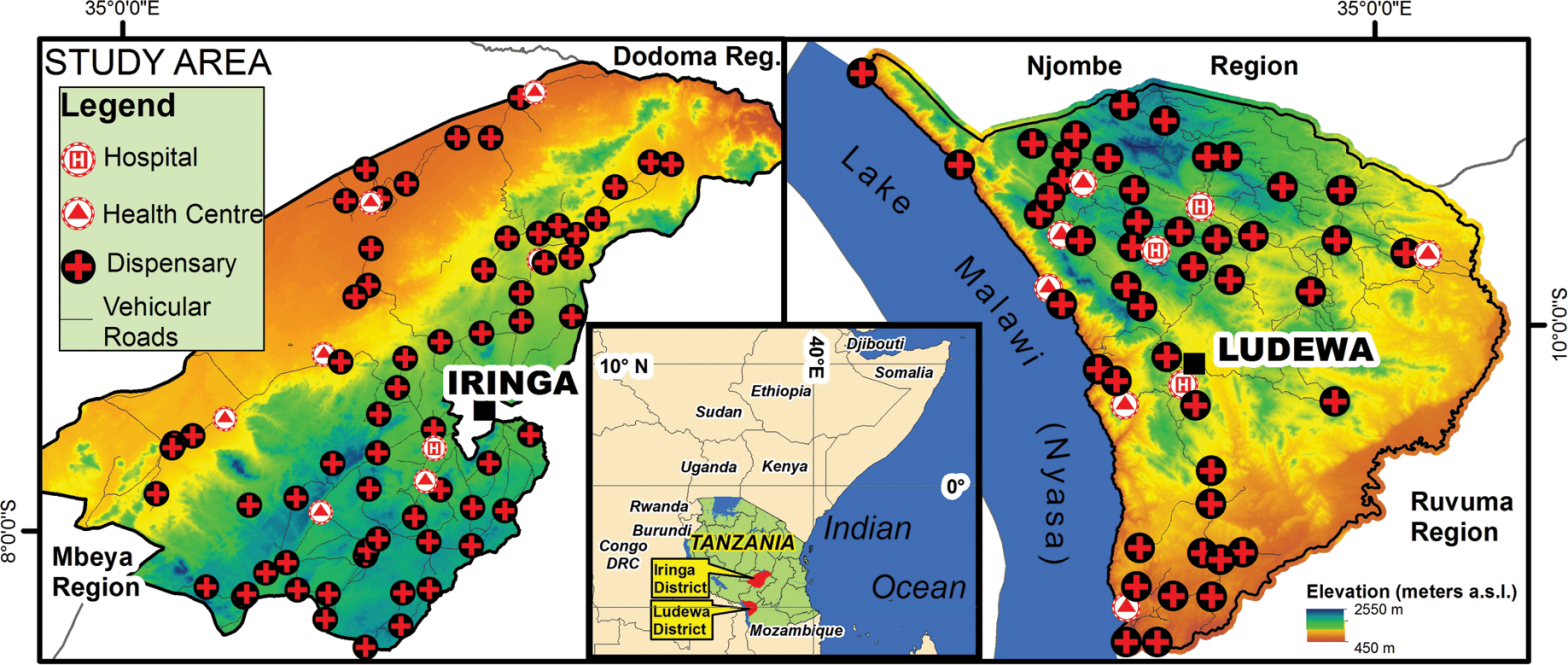
Location map of Ludewa and Iringa Districts.

This study was conducted in two rural districts, Iringa and Ludewa, formerly both part of Iringa Region, and presently in Iringa and Njombe Regions (Fig 1), characterised by high maternal services uptake. According to the latest National Demographic Health Survey the regional estimates for antenatal care coverage (at least one visit) and institutional deliveries were respectively 97.3% and 80.4% [26].

Iringa District has a habitable surface of 9857 Km^2^ and is morphologically and climatically divided into three areas: the Highlands (over 2000 m asl) located in the south-west, the Midlands in the north-east and the Lowlands in the north-west, the latter being extensively covered by a national park and mostly uninhabited. According to the national census the population in 2012 was 254,023, with 85% relying on subsistence farming. The total number of villages was 122 and the road network consisted of 1272 km of tarmac and unpaved roads [28]. Health services in 2012 were available in 72 facilities, of which 65 dispensaries, 6 health centres and one diocesan District-designated hospital. The majority of health facilities were public, with only 27% run by private non-profit organizations. Delivery services were provided by all health facilities except one dispensary located in Ruaha National Park.

Ludewa District is part of Njombe Region and borders Lake Malawi. It has a population of 133,218 and a habitable surface of 6012 Km^2^. The district is predominantly rural with 77 villages and a road network of 970 Km. People derive their livelihood from subsistence farming, fishing, mining and small scale trading [29]. Morphologically Ludewa District is represented in the north-west by a mountainous and rainy area (Livingstone Mountains with an average elevation of over 2000 m asl), in the centre by a midland area with high population density and in the south by a lowland area, mostly flat and fertile near the Ruhuhu river. Health services in 2012 were provided by 55 facilities, of which 46 dispensaries, 6 health centres and 3 hospitals. 20% (11/55) of health facilities were run by private non-profit organizations.

Both districts are characterised by limited road infrastructure and scarce transport services.

### Field data collection

Data on 2012 staffing and health facility deliveries were obtained at district level from the Human Resources Information System and Health Management Information System and were validated during site visits by comparing routinely collected data with local registers. The Iringa District data were part of a larger dataset described in a previous study [18]. Clinicians, enrolled and registered nurses were categorized as skilled birth attendants while nursing assistant were not, according to the health system organization in Tanzania [17]. The lists of villages and existing health facilities were obtained from District Land Offices and District Medical Offices. Geographical data on location of health facilities were collected during site visits from April to August 2013. A waypoint was marked at the main entrance of each facility by using hand-held Global Position System units (Garmin Etrex 10, Nokia E5-00SE) or an USB GPS device connected to a laptop. Longitude and Latitude data were recorded with an accuracy of 5–15 metres in WGS 1984 Datum coordinate system and were converted in WGS_1984_UTM_Zone_36 system.

Geographical coordinates were recorded for all health facilities (55) of Ludewa District, and for 71 out of 72 of Iringa District. As mentioned before, data was not collected from one dispensary that does not provide delivery services.

In Ludewa District more than 650 km of motorable roads and 24 km of footpaths were recorded with the track function of Garmin Etrex GPS respectively by car transport and walking.

### Sources and processing of geographical data

Topographic sheets with scale 1:50000 of United Republic of Tanzania (completed between 1977 and 1982) were used as source of data for villages, watercourses, lakes, roads, tracks and footpaths. Road network and location of villages were updated using remote sensing by comparison of available topographic data with Bing™ and Google Earth™ satellite images. Free online datasets on district boundaries, main road network, main cities, main river network and land use were downloaded from FAO GeoNetwork [30]. A 90 metres cell Digital Elevation Model (DEM) was acquired by US Geological Survey (DEM courtesy of the U.S. Geological Survey) [31] and was used to create the slope and aspect maps. Geographical and administrative data were processed with ArcGIS 10.2™ software (ESRI, Redlands, CA).

### Estimating travel speed

The road network of each district was classified into 5 classes according to road size, type of surface and seasonal condition (Table 1). Road classification was carried out by merging information gathered through Google Earth™ and Bing™ satellite images with 1:50000 maps and data collected in the field. Walking speeds were recorded through a sample walk of 24 km across a morphologically representative area. Data were collected using a healthy male volunteer with a portable GPS. The recorded track log was split into 10% slope intervals and walking speeds were tabulated for each category of slope. The findings from our study were compared with previous published data [32–34]. Despite a similar trend in speed reduction by degree of surface inclination, absolute values were different from those estimated by other authors (Table 2). It was therefore decided to adopt the more conventional Naismith-Langmuir Rule to estimate walking speed for the study population composed mainly of pregnant women (Table 2) [34].

**Table 1:** Road types and travel speeds by motorized and pedestrian transport.

Road Type	Speed km/h
Tarmac	75
Loose Surface	45
Dry Wheater	40
Motorable	20
On Foot	4

**Table 2:** Walking speeds (slope in percent %).

Slope %	Measured Walking Speed (Km/h)	Naismith-Langmuir Rule Speed (Km/h)
< (-)100%	0.61	0.61
(-) 90–100%	0.68	1.28
(-) 80–90%	0.74	1.38
(-) 70–80%	0.98	1.49
(-) 60–70%	1.51	1.63
(-) 50–60%	2.02	1.79
(-) 40–50%	2.54	1.99
(-) 30–40%	3.31	2.24
(-) 20–30%	3.65	3.03
(-) 10–20%	3.82	6.16
(-) 0–10%	3.98	4.30
0%	3.78	4.00
0–10%	3.64	2.99
10–20%	3.51	1.98
20–30%	3.36	1.49
30–40%	3.03	1.19
40–50%	2.72	0.99
50–60%	2.40	0.85
60–70%	2.27	0.75
70–80%	2.14	0.66
80–90%	2.09	0.60
90–100%	2.03	0.54
>100%	1.03	0.24

The Langmuir Rule (1984) assumes a basal speed of 4 km/h and modifies the value according to the following conditions: increased travel time by 0.1 min per 1 m in ascent; reduced travel time by about 0.03 min per 1 m in descent, in the range of slope from -5°: to-12°; increased travel time by about 0.03 min per 1 m in descent for slope steeper than 12° (Noor A.M. et al., 2006).

Vehicular travel time was calculated by applying to each type of road a set of travel speeds as described by other authors (Table 1) [25, 35].

### Population data

Raster datasets on population distribution and population density were obtained from Worldpop Project [36]. Population data for Ludewa and Iringa Districts were updated by applying an adjustment factor to the Worldpop 2010 database based on the 2012 National Census Data [37] without changing the spatial distribution of the population. Data on population density were matched to each spatial model to estimate the percentage of population living in the two-hours’ catchment area.

### Software

Data management and analysis were performed with ArcGIS™ 10.2 for Desktop (ESRI™), and Python scripts compiled by the authors. Raster and network analysis were carried out with ArcGIS™ extensions 3D Analyst, Network Analyst and Spatial Analyst. Geographical conversions and transformations from and to WGS_1984_UTM_Zone_36S (WKID: 32736 Authority: EPSG) were executed by the project tool supplied by ArcGIS™. Topological functions were used to check the consistency and coherence of line data sets. Minor calculations and ancillary data were managed with MS Excel™ spreadsheet functions and Python scripts.

### Spatial analysis

Two spatial models, based respectively on raster and network analysis, were developed to estimate travel time to reach the nearest health facility. Facility catchment area within 2 hours’ walking time was defined with both methods and respective findings were compared for validation. Scenarios with reduced number of delivery sites were described and assessed for population coverage. In addition exploratory network analysis was carried out to assess multimodal transport (pedestrian and vehicular).

#### Raster approach

In the raster method the study area was split into unit cells of 90 square meters, the same resolution used by the Digital Elevation Model. Cost raster maps were created by intersecting basic raster maps with slope and land use surface datasets. Time to cross on foot each cell was adjusted for surface inclination (ascendant or descendant), land use, topographic features and seasonal variation by applying friction coefficients to walking speeds (Table 2 and Table 3). Lakes and swamps were classified as non-passing areas.

**Table 3:** Friction coefficients used to estimate walking time in slope and land use rasters.

Slope %	Coefficient	Dry Season	Coefficient	Wet Season	Coefficient
0–20%	0.98	Open Areas	0.95	Open Areas	1.00
20–40%	1.00	Bushy Areas	1.00	Bushy Areas	1.05
40–60%	1.20	Forest Areas	1.05	Forest Areas	1.10
60–80%	1.04				
80–100%	1.06				
>100%	1.08				

Friction coefficients were applied to each raster cell to estimate the time needed to cross the cells on foot according to surface characteristics.

Walking time towards health facilities was estimated with the Path Distance ArcGIS^TM^ Tool by identifying the shortest distance to the nearest health facility. Total walking time was estimated by adding up the time needed to cross contiguous cells and by taking into consideration geographical obstacles and slow crossing areas.

Cost distance maps were built for two scenarios: one with all existing health facilities and one with a reduced number of facilities that could be accessible within two hours’ travel time by approximately 80% of the population. Selection criteria for including health facilities in the second scenario were based on spatial aspects and population density.

The Path Distance Output Raster was matched with population data (AfriPop) to estimate the cumulative percentage of population living within subsequent 20 minutes’ intervals of walking time. The 20 minutes’ time interval was arbitrarily chosen to map consecutive catchment areas around each health facility to provide sufficient details at local level. To compare population coverage by different scenarios three levels of access to health facilities were considered: less than one hour, less than two hours and greater than 2 hours’ walking time. These thresholds were based on previous studies [38, 39] and on general guidelines [21]. The analysis was performed with the function Zonal Statistic as table tool of ArcGIS™.

#### Network approach

In rural settings such as in Iringa and Ludewa Districts, where road infrastructure and vehicular transport is limited, the application of network-based methods is hampered by lack of sufficient digital data in vector form. To overcome this constraint we constructed a network based on a combination of real and virtual data. Existing road network was merged with a virtual hexagonal /triangular mesh of lines. The surface of Iringa and Ludewa Districts was divided into 342000 and 539000 hexagonal / triangular areas with side lengths up to 223 metres.

Each side is characterized by an attribute value of estimated travel time towards the health facilities, according to type of slope (ascendant or descendent) and mode of transport (pedestrian or vehicular). A similar technique was previously applied in urban areas of Dar es Salaam [40]. As validation, findings from network analysis were compared with data recorded during the sample walk. Route analysis methods were applied to the virtual network to draw the shortest track between the starting and ending point of the sample walk.

For each health facility catchment areas based on consecutive 20 minutes’ walking time were built. Cumulative population coverage within the three levels of access was estimated with a Zonal Static Tool matched with the Afripop Raster Database. A scenario with a reduced number of delivery sites was produced for pedestrian transport and was compared with the raster methods outputs for validation. An additional scenario for both pedestrian and vehicular transport (multimodal transport) was produced as an attempt to simulate travel patterns with motorised transportation.

### Ethical considerations

Data used in this study were either available as unrestricted sources in the public domain or provided with permission from local health authorities. Health information was extrapolated from routinely collected data in an aggregated form. No data was collected at individual level.

## Results

### Maternal health services

Delivery services in 2012 were provided by all levels of health facilities in Iringa and Ludewa Districts.

Data on 2012 institutional deliveries were available for 69/71 (97.2%) health facilities in Iringa District and for 48/55 (87.3%) in Ludewa District. Out of 7645 reported institutional deliveries of Iringa District, 2140 (28%) took place at the only hospital offering comprehensive emergency obstetric services (CEmOC), while in Ludewa District a total of 2808 deliveries out of 4089 (68.7%) reported institutional deliveries were carried out in the three hospitals of the district that provide CEmOC. Remaining institutional deliveries were scattered across first-line health facilities, respectively 5505 deliveries in 70 health facilities in Iringa and 1281 deliveries in 52 health facilities in Ludewa District. Yearly caseload was less than 100 deliveries in 48/70 (68.6%) first-line health facilities in Iringa District and 43/52 (82.7%) first-line health facilities in Ludewa District (Table 4). Median delivery caseload for first-line health facilities in Iringa District was 65 (range 2–277) and 22 (range 2–117) in Ludewa District.

**Table 4:** Staffing level in first-line health facilities (dispensaries and health centres). Iringa and Ludewa districts. 2012.

*Number of skilled birth attendants*	*Iringa District health facilities*		*Ludewa District health facilities*	
	*n*	*(%)*	*n*	*(%)*
0	2	(2.9)	12	(23.1)
1	30	(42.9)	29	(55.8)
≥ 2	38	(54.3)	11	(21.2)
Total	70		52	

Data on staffing were available for all health facilities that provide delivery services. We found that 2/64 (3.1%) dispensaries in Iringa District and 12/46 (26%) dispensaries in Ludewa District had no skilled birth attendants and about half of first-line health facilities were staffed with only one SBA, 30/70 (42.9%) in Iringa District and 29/52 (55.8%) in Ludewa District (Table 5).

**Table 5:** Delivery caseload in first-line health facilities (dispensaries and health centres). Iringa and Ludewa districts. 2012.

*Number of deliveries*	*Iringa District health facilities*		*Ludewa District health facilities*	
	*n*	*(%)*	*n*	*(%)*
0–49	21	(30.0)	41	(78.8)
50–99	27	(38.6)	2	(3.8)
100–199	17	(24.3)	2	(3.8)
≥ 200	3	(4.3)	0	(0.0)
NA*	2	(2.9)	7	(13.5)
Total	70		52	

* NA: data not available.

### Geographical coverage of maternal services

#### Raster approach

**Fig 2:**
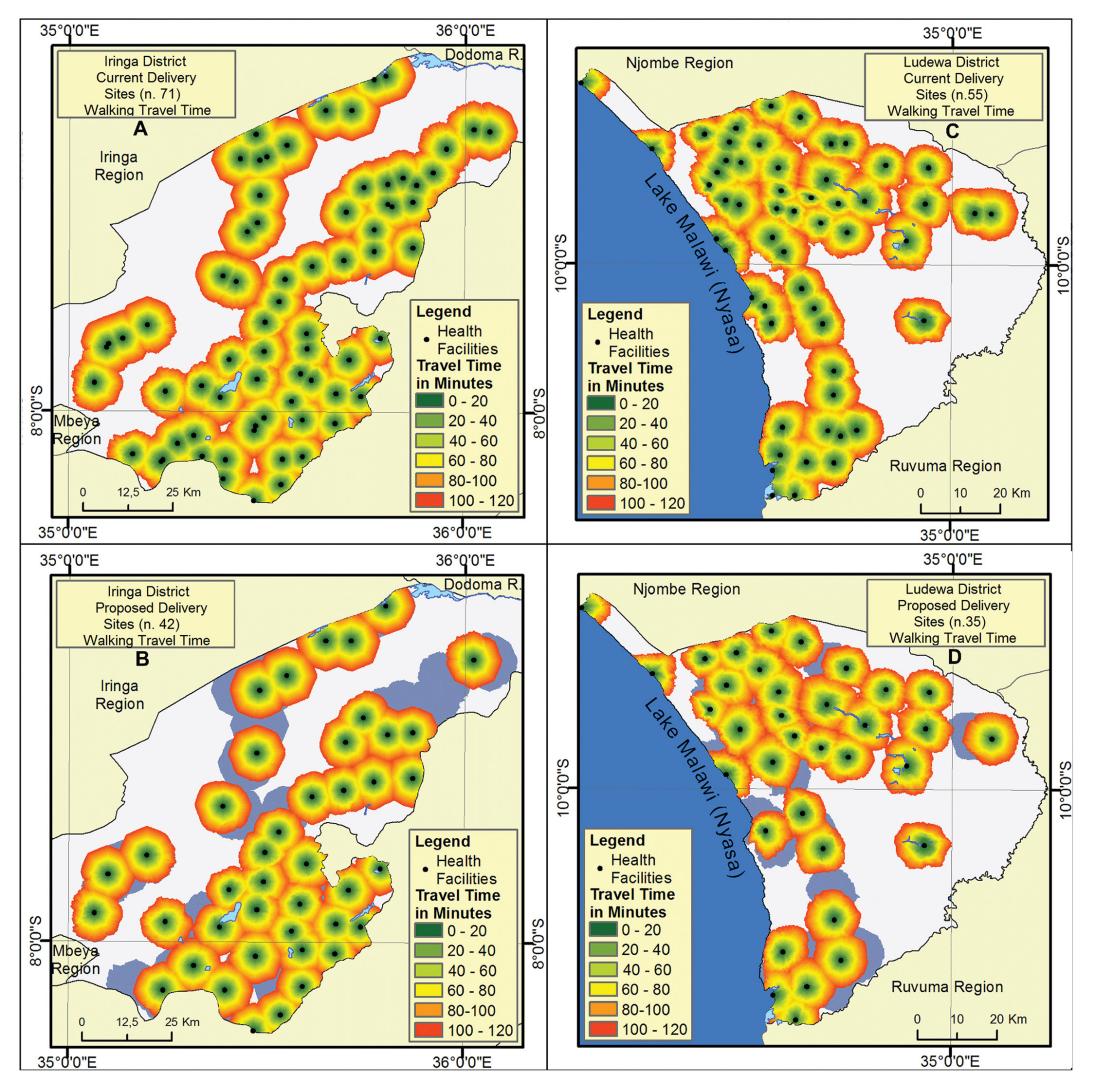
Catchment area estimated by raster analysis. The areas around health facilities represent a 2 hours’ catchment divided in 20 minutes’ intervals. (A) Iringa District current scenario with all delivery sites; (B) Iringa District proposed scenario with reduced number of delivery sites; (C) Ludewa District current scenario with all delivery sites; (D) Ludewa District proposed scenario with reduced number of delivery sites. The grey shades delimit the areas that will loose accessibility within 2 hours by a 40% reduction of delivery sites.

Geographical coverage of health facilities in terms of walking time is displayed in Fig 2A and 2C. Various overlapping areas are detected among several health facilities, especially for walking distances between one and two hours.

Travelling distances are summarized in Table 6. At present 88% and 82% of women in Iringa District and Ludewa District reside within 2 hours’ walking time to a facility offering delivery care. A reduction of number of delivery sites from 71 to 42 (40.8% reduction) in Iringa District and from 55 to 35 (36.4% reduction) in Ludewa District would decrease by 7% the proportion of women living within two hours’ travel time and only few areas of the districts, displayed as grey shaded in Fig 2B and 2D would face an increased access time beyond 2 hours.

**Table 6:** Population coverage by walking distance to health facilities. Present scenario and projections for reduced number of delivery sites. Raster analysis.

	Population coverage (%)			
Walking time	*Iringa District*		*Ludewa District*	
	Current delivery sites (N = 71)	Proposed delivery sites (N = 42)	Current delivery sites (N = 55)	Proposed delivery sites (N = 35)
≤ 1 hour	54.1	41.1	39.9	26.1
≤ 2 hours	87.8	83.0	82.3	76.8
> 2 hours	12.2	17.0	17.7	23.2

#### Network approach

**Fig 3:**
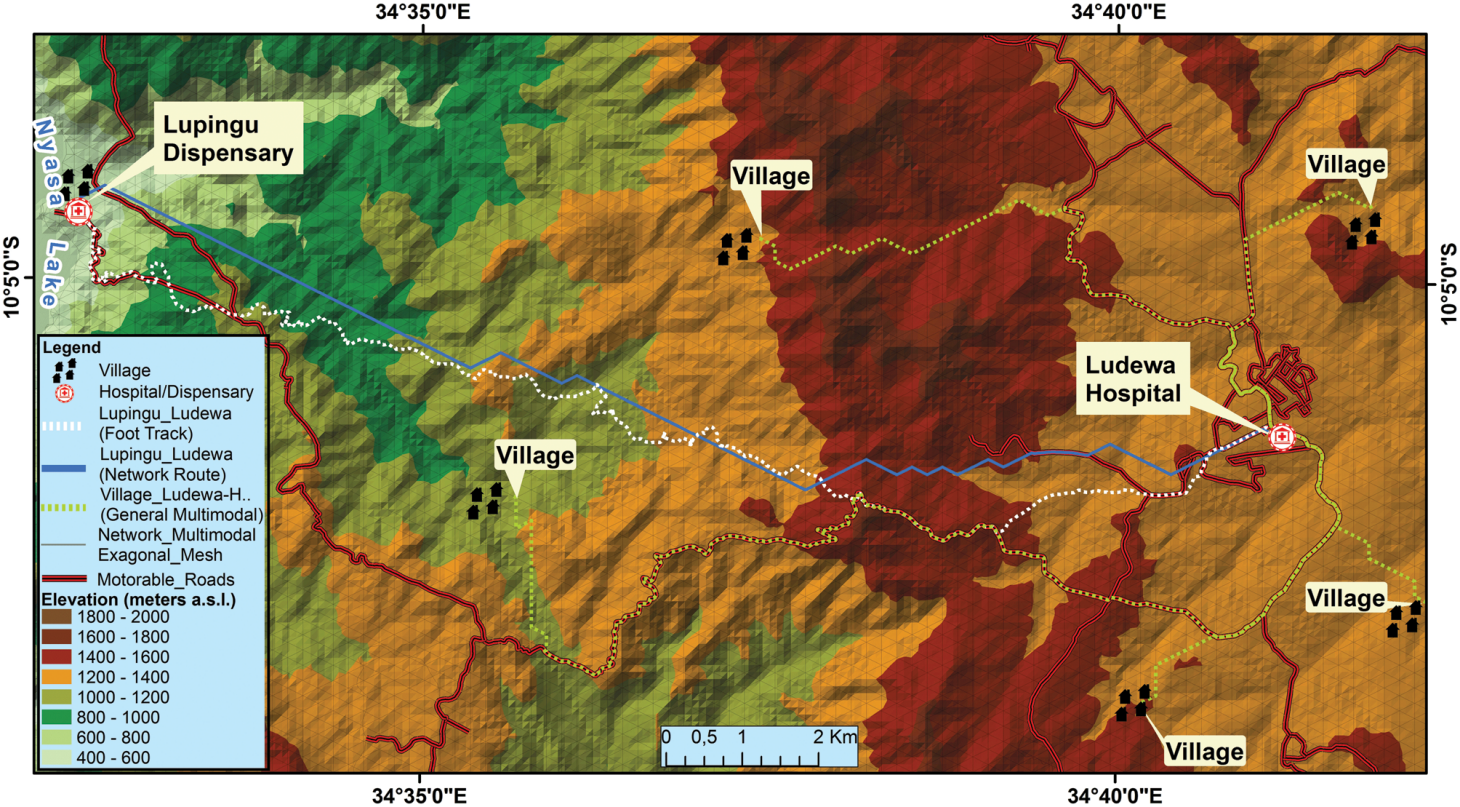
Outputs from sample walk and network analysis. The route covered by the volunteer (white dashed) corresponds to the trajectory traced by the software on the virtual network (blue line). Other tests are relative to four villages set at few kilometres away from motorable roads. The multimodal output (yellow dashed) automatically estimates the faster route to the hospital and is based both on virtual mesh lines and on existing motorable roads (red lines).

Findings from sample walk and network analysis are displayed in Fig 3. The route covered by the volunteer (white dashed) is partially overlapping with the trajectory traced on the virtual network by the application Route Analysis of ArcGIS™ (continuous blue line). Although in some areas shorter distances are automatically selected by the software, overall time to reach the nearest health facility is similar to that recorded by the volunteer (5 hours and 30 minutes estimated by the software versus 5 hours and 38 minutes recorded in the field). This can be explained by the more conventional walking speeds applied to the network analysis (Naismith-Langmuir Rule) compared to the walking pace of the enrolled walker. There is also evidence of validity for the multimodal transportation model. The software estimates the shortest travel time needed to reach health facilities by combining vehicular transport on motorable roads to pedestrian speeds on virtual network (Fig 3 red and dashed yellow lines).

**Fig 4:**
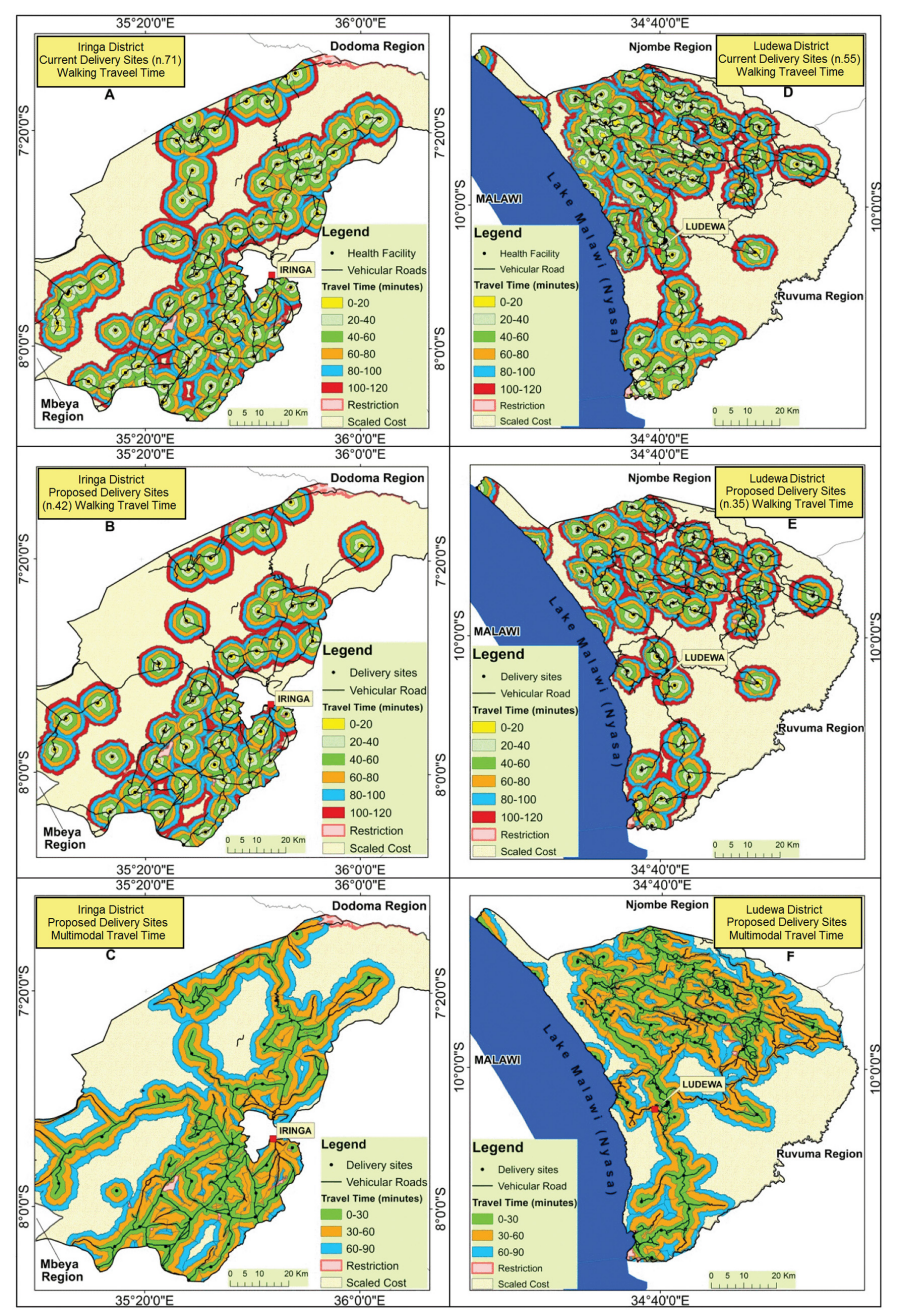
Catchment area estimated by network analysis. The areas around health facilities represent a 2 hours’ catchment divided in consecutive intervals for walking speed and for multimodal transport in Iringa and Ludewa Districts. (A, D) Current scenario with all delivery sites; (B, E) reduced number of delivery sites using walking speed; (C, F) reduced number of delivery sites using multimodal transport (vehicular and walking speed). Restriction: non-passing areas (lakes, swamps, etc.). Scaled cost: areas beyond 2 hours’ travel time.

After validation, network analysis was applied to both districts to define areas located within 2 hours’ travel time from each health facility (Fig 4A and 4D). Several overlapping areas are observed at one hour walking time and above, as has already been highlighted with raster method. To minimize overlap across health facilities a scenario with a reduced number of delivery sites is reproduced both for pedestrian (Fig 4B and 4E) and for multimodal travel time (Fig 2C and 4F).

Concerning population coverage, again, results from network analysis (Table 7) are consistent with findings from raster methods (Table 6). Both approaches suggest that a substantial decrease of number of delivery sites translates into less than 10% reduction of population within 2 hours’ walking time.

**Table 7:** Population coverage by walking distance to health facilities. Present scenario and projections for reduced number of delivery sites. Network analysis.

	Population coverage (%)			
Walking time	*Iringa District*		*Ludewa District*	
	Current delivery sites (N = 71)	Proposed delivery sites (N = 42)	Current delivery sites (N = 55)	Proposed delivery sites (N = 35)
≤ 1 hour	51.4	38.8	38.0	24.6
≤ 2 hours	87.6	82.1	81.4	75.6
> 2 hours	12.4	17.9	18.6	24.4

## Discussion

Our analysis indicates that about half of the first-line health facilities were inadequately staffed and had a yearly case load below 100 deliveries limiting their ability to provide quality delivery care. Reducing the number of delivery sites by 40%, taking into consideration geographical and demographical aspects, would lead to an increased access time beyond 2 hours for only 7% of population. To the best of our knowledge this is the first study on delivery services reorganization based on travel time in a rural context. A concentration of delivery sites could help to address the dilemma of accessibility and quality of care which resource poor settings face. The reduction of delivery sites would allow to concentrate skilled staff and obstetric services in higher volume settings with the aim of improving childbirth care, as recently recommended by other authors [18,19,41].

The investigated area is characterized by high health facility density and high institutional delivery coverage and offers therefore a paradigm to study the effects of an increased number of facilities on childbirth services’ in resource-limited contexts. High geographical coverage was observed, with over 82% of population living within 2 hours’ walking time from a health facility, yet half of first-line facilities were unable to provide skilled attendance at birth 24h 7 days/week due to low caseload and insufficient staff. Our findings are consistent with previous studies from rural Tanzania where a dilemma between high health facilities coverage and quality of obstetric services has been highlighted [17–19, 42, 43].

### Reduced number of delivery sites

The distinction between access to a service site and access to effective case management is of paramount importance [44], particularly as densification of health facilities in resources limited settings is implemented.

Although shorter distance to point of care has been related to reduced under five child mortality in rural Tanzania, following improved access to immunization and to basic treatments [45–47], no association was found either between distance to health facility and early neonatal mortality [6] or between distance to any health facility and maternal mortality in similar rural contexts [48]. These apparently contrasting findings can be attributed to intrinsic differences between childbirth care and other preventive and curative interventions. The former requires functioning health facilities adequately equipped and staffed round the clock while other services do not require full time activity.

Poor staffing and low caseload in peripheral health facilities have already been described in rural settings [17, 18]. Our data suggest that in remote areas the picture is even more grim, with over a quarter of first-line health facilities not having any qualified staff and half having only one skilled birth attendant, who is unable to cover 24 hours shifts 7 days/week.

Policies aimed to improve population coverage by reducing distance might in fact affect the quality of services by diluting scarce resources. According to Tanzanian national policy, a further 3088 dispensaries, 2074 health centres and 19 district hospitals will be established by 2017 [11], meaning the number of first-line facilities will double over the next few years. As a consequence, the severe shortage of human resources in Tanzania, currently estimated at 70% [49], will increase and institutional deliveries will be dispersed over a vast number of scantily staffed and poorly equipped peripheral health facilities. Poorer women will pay the toll as they cannot afford to bypass first-line health facilities for delivery [18].

Although the study did not aim to investigate quality of peripheral childbirth services directly, we assessed two aspects of first-line facilities that are essential prerequisites to provide quality of care: availability of skilled staff and case volume. The importance of human resources in the quality of emergency obstetric care and in reducing maternal mortality has been extensively described [50]. Staff shortage and inadequate training have been reported as major barriers to timely and appropriate obstetric care at facility level [51]. Beyond training, caseload is an important factor to maintain skills and to improve outcome quality. In several European countries minimum caseload requirements have been set for surgical procedures to improve patients outcome and there is evidence of association between obstetrical volume and early neonatal mortality [52]. Caseload is particularly relevant to identify BEmOC facilities as a set of signal functions has to be performed at least once every three months [14]. Given the rare onset of obstetrical complications, a large number of deliveries is necessary for each BEmOC facility to perform periodically all signal functions.

Previous studies have addressed the quality gap in first-line health facilities [15, 17,18, 53] and some authors have recommended providing obstetric care only in high-volume settings and not unconditionally at first level [8, 18]. Different solutions have been put forward, such as concentrating deliveries in health centres [19] or offering delivery services at second-line primary health care units [54]. Our data show that locating potential delivery sites with the support of GIS-methods instead of using mere demographic and administrative criteria allows to take into consideration travel time and to ensure physical access. In practice, this could not be implemented exclusively on geographical considerations, but would require discussion with all relevant local stakeholders and involvement of community leaders to avoid decreased utilization of childbirth services.

### Use of network methods in rural areas

Network analysis can provide a reliable picture of women mobility in rural areas. Compared to raster methods where groups of contiguous pixels are considered, network methods can reproduce existing roadmaps and will allow more accurate analysis.

From a methodological point of view our results indicate that network analysis based on real and virtual data allows flexible and accurate simulations in rural contexts and can be adapted to changing scenarios as transport system is developing and travel pattern of pregnant women will change over time.

Findings from network analysis may suggest where to locate resources, such as public transport, ambulances and maternity waiting homes to ensure access to delivery sites for women living beyond two hours [53, 55].

### Strengths and limitations

The main strength of the study is the amount and detail of geographical data collected both in the field and by remote sensing. Furthermore, distance was estimated taking into consideration geographical barriers and was measured as travel time which is more relevant to obstetric services than conventional metric measures. Although straight-line (Euclidean) distance can be reasonably used as a proxy for potential spatial access in flatter areas [56], raster or network methods are more suitable to estimate travel time in mountainous contexts and can supply adequate details for planning at local level.

The lack of official digital topographic maps was a constraint to our study. Road network data were manually updated by digitalizing satellite images from Google Earth™ and Bing but were not always consistent with data provided by local authorities. As routine data are usually less accurate and outdated, we opted for actual data from the field or from satellite images without further opportunity to validate the topographic findings. When updated official digital topographic maps of this area will be available the accuracy of our network method in modelling mobility and travel time will improve.

Another possible limitation might be the arbitrarily chosen 2 hours’ walking time cut-off. Although the existing literature does not provide clear standards for maximum acceptable travel time to reach obstetric facilities, a 2 hours’ interval is commonly considered sufficient and is in line with available guidelines on childbirth services [21] and previous GIS-based studies [38, 39, 51, 53].

Despite the limited geographical size, the investigated area provides essential information for other rural settings that are planning to increase the health facility numbers with the aim of improving institutional childbirth coverage.

## Conclusions

This study indicates that in a rural high coverage context a 40% reduction of delivery sites will lead to a 7% loss of geographical access. Such careful reduction of delivery sites using GIS modelling methods has the potential to assist decision makers on where to concentrate scarce resources by creating higher volume settings.

Although a small percentage of the population will suffer an increase in distance to health facilities, a policy change in the organization of obstetric services might provide overall improved childbirth care, particularly for the rural poor who preferentially use first-line facilities.

Further research should aim to investigate the effects of the proposed policy adjustment in a limited geographical area. In particular, the effect of fewer strengthened delivery sites on maternal and newborn mortality should be assessed, and whether the loss of proximity affects institutional delivery coverage.

## Supporting Information

S1 Dataset Ludewa District health facilities dataset. Staffing level and number of deliveries. 2012. Ludewa District, Tanzania.
(XLS)

S2 Dataset Iringa District health facilities dataset. Staffing level and number of deliveries. 2012. Iringa District, Tanzania.
(XLS)
